# Intraoperative collection of autologous platelet-rich plasma from the cardiopulmonary bypass circuit upon initiation of extracorporeal circulation

**DOI:** 10.1186/s13019-020-01388-5

**Published:** 2021-02-05

**Authors:** Takeshi Honda, Yuji Kanaoka, Hiroshi Furukawa, Taishi Tamura, Noriaki Kuwada, Takahiko Yamasawa, Yoshiko Watanabe, Yasuhiro Yunoki, Atsushi Tabuchi, Kazuo Tanemoto

**Affiliations:** Department of Cardiovascular Surgery, Kawasaki Medical School, 577 Matsushima, Kurashiki, Okayama, Japan

**Keywords:** Platelet, Cardiopulmonary bypass, Preoperative autologous platelet collection, Platelet function, Cardiac surgery

## Abstract

**Objectives:**

The aim of this study is to evaluate the possibility of the autologous platelet-rich plasma (PRP) collection from the cardiopulmonary bypass (CPB) circuit and to evaluate its effect on the aggregative function.

**Methods:**

For seventy-two patients undergoing cardiac surgery with CPB, an autologous PRP was prepared using the Haemonetics Component Collection System® by drawing blood from the CPB circuit immediately after CPB was established. The blood samples were taken at three points for examination, A: beginning of surgery, B: immediately after heparin reversal with protamine following discontinuation of CPB, C: after the collected autologous PRP was returned to the patient. Platelet count and platelet aggregation ability were analyzed.

**Results:**

The mean platelet count in autologous PRP was 5.5 (range: 3–14) units. Platelet count decreased by 115.0 (±27.3) × 1000/μl from A to B and increased by 27.3 ± 17.2 (× 1000/μl) from B to C.

When platelet aggregation was measured by Adenosine Diphosphate (ADP) 3.0 μM, it decreased by 42.6% ± 12.1% from A to B and increased by 8.7% ± 7.4% from B to C.

**Conclusions:**

Autologous PRP can be safely collected by drawing blood from the CPB circuit, platelet count and aggregation ability significantly decreased after CPB including autologous PRP collection. Some improvement was detected in the number of the platelets count and platelet aggregation ability by administrating an autologous PRP even if autologous PRP is collected from CPB circuit.

**Trial registration:**

UMI-CTR, UMIN000023776. Registered 1 October 2016.

## Introduction

Intraoperative thrombocyte impairment occurs in cardiac surgery due to exposure of blood to the cardiopulmonary bypass circuit; intraoperative thrombocyte impairment affects both thrombocyte count and function [1]. However, there is no established solution for this problem. Notably, platelet impairment can be treated by homologous platelet transfusion; however, platelet products tolerate a brief storage period, such that future supply may be difficult to maintain [2]. Preoperative autologous platelet-rich plasma (PRP) collection represents a potential solution, but the current method for autologous PRP collection requires approximately 90 min [3]. It is ideal to perform autologous PRP collection before the administration of heparin and establishment of cardiopulmonary bypass (CPB), preoperative PRP collection delays the progress of surgery.

There have been no reports of the impact on aggregation ability of platelets collected during heparin administration immediately after the initiation of CPB. In a previous study, we confirmed that there is no difference in the aggregation ability of autologous platelets collected before and after administration of heparin and that platelet count and platelet function of PRP are not affected by heparin administration in animal experiments [4]. Based on these findings, the aim of this study is to investigate the feasibility and usefulness of autologous platelet collection from the CPB circuit after administration of heparin in clinical cases. Autologous platelet transfusion under administration of antiplatelet drug is feasible. However, because platelet aggregation ability is used to evaluate the effectiveness of autologous platelet transfusion, the patients with administration of antiplatelet drug were excluded from this study.

### Subjects

**Fig. 1:**
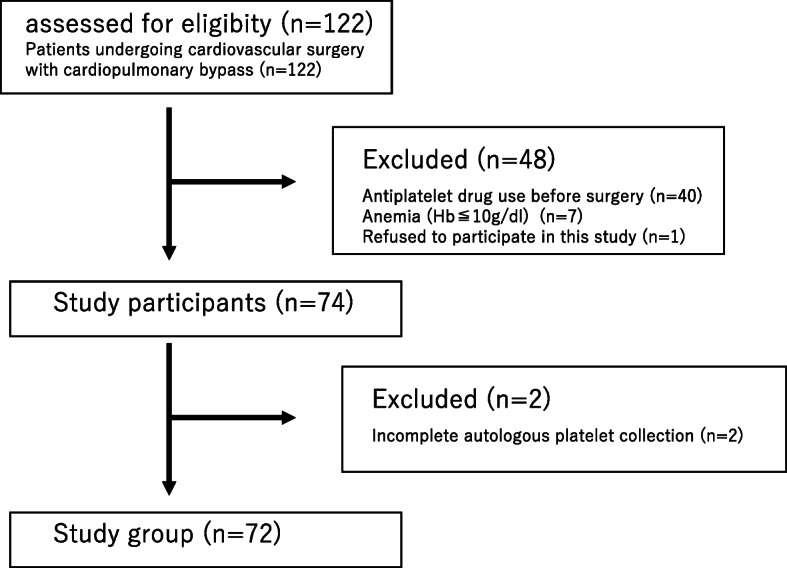
The study flow diagram of this study

This study examined one hundred and twenty-two patients who underwent cardiovascular surgery with CPB at Kawasaki Medical School Hospital, from November 2016 through December 2018. Patients were excluded if they met the following criteria: 1. they did not provide consent; 2. a drug that affects platelet function was administered before surgery; 3. they showed a high degree of anemia (Hb ≤ 10 mg/dl). In the excluded patients, forty were for the preoperative antiplatelet drug use mainly aspirin, seven for anemia, and one for the refusal of the patient and its family. In addition, two patients were excluded due to problems with the blood collection procedure. In all cases, the clinical study plan was explained to the patients, and the number of the subjects in this study was seventy-two (Fig. 1). This clinical study was approved by the ethics committee of Kawasaki Medical School (2478–2).

## Methods

**Fig. 2:**
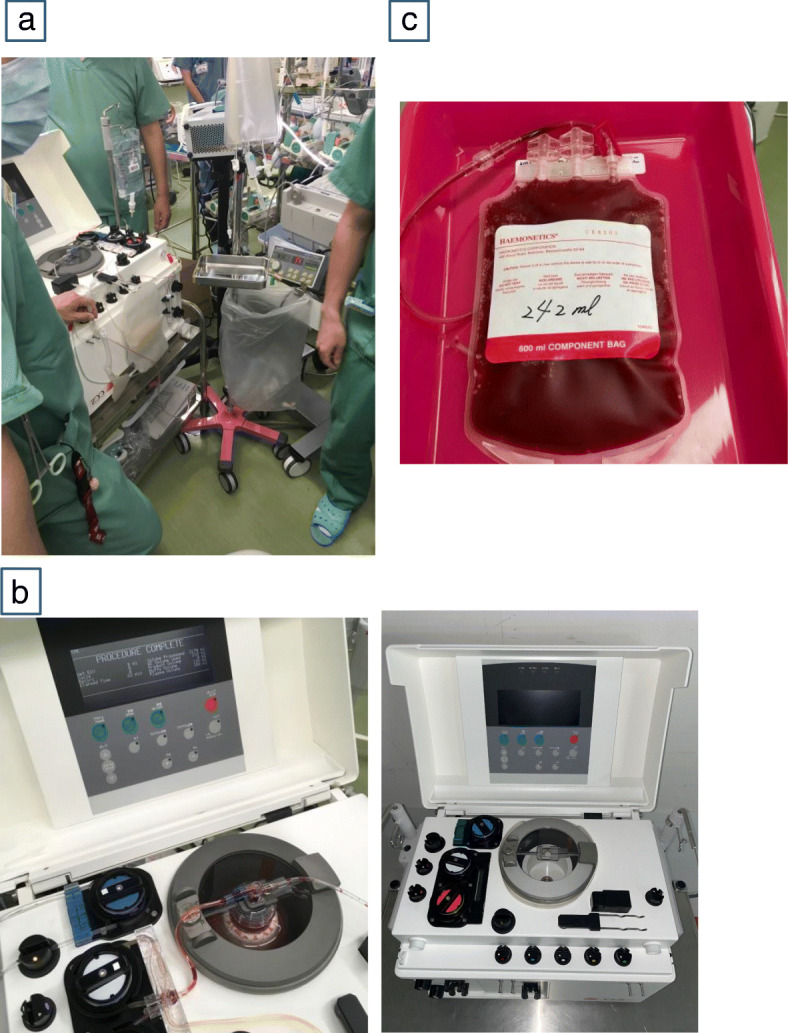
The process of autologous platelet-rich plasma collection. **a**: Haemonetics Component Collection System® (Haemonetics, Braintree, MA, USA) is connected to the cardiopulmonary bypass circuit. **b**: Platelets separation started upon initiation of cardiopulmonary bypass. **c**: Autologous platelet-rich plasma is returned to the patient intravenously after discontinuation of cardiopulmonary bypass and administration of protamine

Cardiopulmonary bypass was initiated after administration of heparin at the dose of 300 U/kg. By drawing blood from the CPB circuit, PRP was prepared as an autologous platelet product using the Haemonetics Component Collection System® (Haemonetics Corporation, Boston, MA, USA) before platelet function was damaged due to prolonged CPB (Fig. 2). Following discontinuation of CPB, autologous PRP was returned to each patient after protamine administration.

**Fig. 3:**
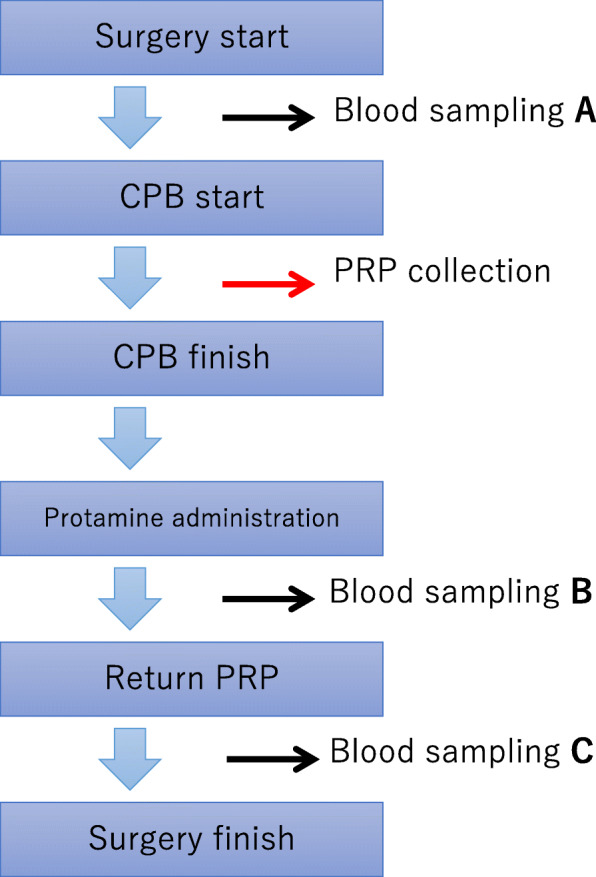
Collection of platelet-rich plasma and timing of blood sampling. Blood sampling was conducted at each of the following time points: **a**: beginning of surgery (before administration of heparin), **b**: after discontinuation of cardiopulmonary bypass and protamine administration; and **c**: after returning the autologous platelet-rich plasma. Platelet count and platelet aggregation ability (MCM Hema Tracer 712, MC medical, Tokyo, Japan) were measured

Blood sampling was conducted at each of the following three points, A: beginning of surgery before heparin administration, B: discontinuation of CPB and immediately after protamine administration; and C: after returning autologous PRP to the patient. Using these samples, platelet count and platelet aggregation ability (MCM Hema Tracer 712, MC medical, Tokyo, Japan) were measured (Fig. 3). Platelet aggregation-inducing substances were 1.0 μM ADP, 3.0 μM ADP, 0.25 μg/ml collagen, and 2.0 μg/ml collagen. Significant differences were assessed by comparing blood from each of the time points using the Student’s t-test. A *p* value of < 0.05 was considered to be statistically significant. All analyses were performed using SPSS Ver. 20.0 (IBM SPSS; Armonk NY, USA).

## Results

In total, seventy-two patients were included. Mean patient age was 72.4 ± 12.9 years. The male/female ratio was 49:23. The types of surgery consisted of forty-two cases of valvular disease surgery, twenty-six cases of aortic aneurysm surgery, and four cases of coronary artery bypass surgery + others. The mean operation time was 378.6 ± 141.8 min, and the mean number of platelets at the initiation of surgery was 182.3 ± 31.6 × 1000/μl (Table 1). The mean platelet count in autologous PRP was 120.8 ± 69.3 × 10^11^, corresponding to approximately 5.5 units (Table 2).

**Table 1:** Patient characteristics and operative data

Factors (***n*** = 72)	Mean	SD
Age (years)	72.4	12.9
Male/Female	49:23	
Height (cm)	157.8	8.6
Weight (kg)	61.0	13.6
Body mass index	23.7	4.2
Body surface area (m^2^)	1.62	0.20
Procedure (*n* = 72)		
Aortic	26	
Valvular	42	
Coronary + others	4	
Operation time (min)	378.6	141.8
Extracorporeal circulation time (min)	129.3	44.6
Aortic cross-clamp time (min)	93.6	33.0
Selective cerebral perfusion time (min) (*n* = 20)	**68.7**	**41.3**
Lowest temperature (°C)	32.7	3.5
Drain bleeding within 24 h postoperatively (ml)	551.6	353.0
Reoperation		0

**Table 2:** Platelet count and maximum aggregation ability

Factors	Mean	SD
Collected platelet-rich plasma (ml)	233.3	43.5
Buffy (ml)	128.3	34.0
Plasma (ml)	104.5	18.6
Number of platelets in platelet-rich plasma (×10^11^)	120.8	69.3
Number of platelets in A blood (×1000/μl)	182.3	31.6
Number of platelets in B blood (× 1000/μl)	63.5	30.5
Number of platelets in C blood (×1000/μl)	93.4	43.1
Maximum aggregation rate in A blood		
1.0 μM ADP (%)	54.7	17.6
3.0 μM ADP (%)	68.9	13.0
0.25 μg/ml collagen (%)	40.5	25.3
2.0 μg/ml collagen (%)	72.7	14.5
Maximum aggregation rate in B blood		
1.0 μM ADP (%)	17.4	5.8
3.0 μM ADP (%)	25.4	10.8
0.25 μg/ml collagen (%)	10.5	4.2
2.0 μg/ml collagen (%)	28.5	16.9
Maximum aggregation rate in C blood		
1.0 μM ADP (%)	21.7	9.5
3.0 μM ADP (%)	34.1	14.1
0.25 μg/ml collagen (%)	9.5	2.9
2.0 μg/ml collagen (%)	38.1	19.6

A: Beginning of surgery (before administration of heparin)

B: After discontinuation of cardiopulmonary bypass and protamine administration

C: After returning autologous platelet-rich plasma

**Fig. 4:**
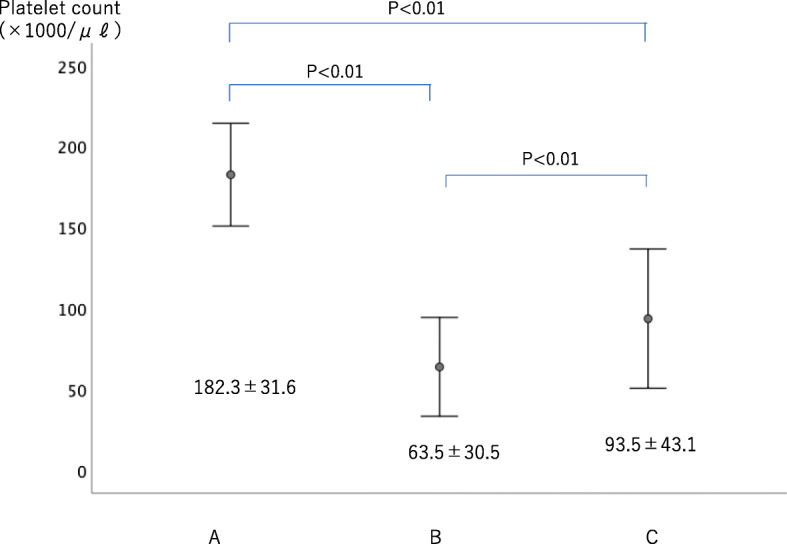
Change of platelet count during surgery. The platelet count significantly decreased after CPB including autologous PRP collection. A significant increase in platelet count was observed upon returning autologous platelet-rich plasma to the patients

**Fig. 5:**
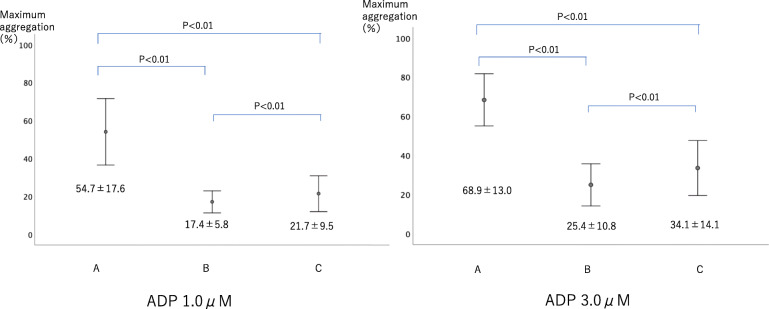
Change of platelet aggregation ability using ADP (Adenosine diphosphate). Maximum aggregation rate increased significantly for 1.0 μM ADP, 3.0 μM ADP, 2.0 μg/ml

**Fig. 6:**
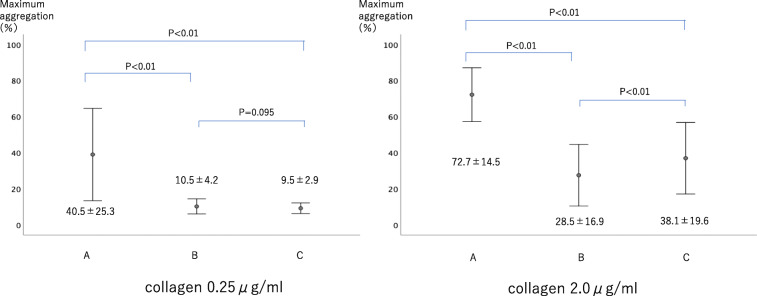
Change of platelet aggregation ability using collagen. Maximum aggregation rate increased significantly for collagen 2.0 μg/ml. However, an increase in maximum aggregation rate was not observed in 0.25 μg/ml collagen due to its low reagent concentration

From point A to point B, the reduction in platelet count was 118.3 ± 31.4 × 1000/μl. The increase in platelet count from point B to point C was 27.3 ± 17.2 × 1000/μl (Fig. 4). The transition of maximum agglutination ability was measured by four methods depending on the aggregation-inducing agent. When using ADP 1.0 μM, maximum agglutination ability decreased by 36.9 ± 15.9% from point A to point B and increased by 4.3 ± 7.8% from point B to point C. When using ADP 3.0 μM, maximum agglutination ability decreased by 36.9 ± 15.9% from point A to point B and increased by 8.7 ± 7.4% from point B to point C. When using collagen 0.25 μg/ml, maximum agglutination ability decreased by 29.3 ± 24.1% from point A to point B and decreased from point B to point C by 1.0 ± 2.8% (Fig. 5). When using collagen 2.0 μg/ml, maximum agglutination ability decreased by 44.0 ± 15.9% from point A to point B and increased by 9.6 ± 9.4% from point B to point C (Fig. 6).

We analyzed the results of 46 patients with normothermic valvular/CABG operation (normothermic valvular surgery group) and 26 patients with hypothermic aortic operation (hypothermic aortic surgery group) separately. Operative time, CPB time and cross clamp time were significantly longer in the hypothermic aortic surgery group, and lowest body temperature was significantly lower in hypothermic aortic surgery group. Moreover, the ratio that needed a platelet blood transfusion was significantly higher in hypothermic aortic surgery group. However, no significant differences were observed between the groups in terms of blood loss volume after operation (Table 3).

**Table 3:** Clinical outcomes in normothermic valvular operation and hypothermic aortic operation

	Normothermic valvular surgery	Hypothermic aortic surgery	*p* value
Number of patients	46	26	–
Age	71.4 ± 14.3	73.8 ± 9.6	0.402
Gender (male)	29 (63.0%)	20 (80.1)	0.095
BSA(m^2^)	1.61 ± 0.22	1.67 ± 0.15	0.274
Platelet count(× 10^4^)	19.1 ± 4.3	17.2 ± 2.8	0.725
Operative time (min)	327.7 ± 80.6	464.2 ± 173.6	*P* < 0.05
CPB time (min)	149.0 ± 40.7	242.5 ± 89.2	*P* < 0.05
Clamp time (min)	80.4 ± 10.8	126.8 ± 44.5	*P* < 0.05
Body temperature(°C)	34.7 ± 0.6	27.9 ± 3.4	*P* < 0.05
Blood loss after surgery (ml)	522.3 ± 326.7	589.0 ± 395.1	0.48
Re-thoracotomy for bleeding	0	0	1.0
Homologous platelet transfusion	12/46 (26.1%)	21/26 (80.8%)	*P* < 0.05

**Fig. 7:**
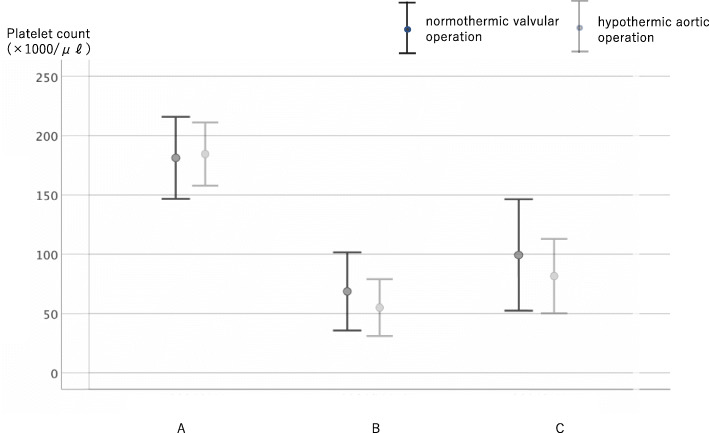
Change of platelet count during surgery in normothermic valvular operation and hypothermic aortic operation. No significant differences were observed between the groups in terms of platelet count during surgery

**Fig. 8:**
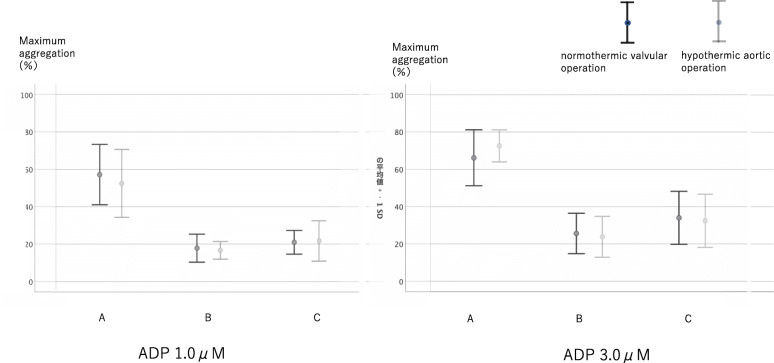
Change of platelet aggregation ability using ADP in normothermic valvular operation and hypothermic aortic operation. No significant differences were observed between the groups in terms of platelet aggregation ability during surgery

**Fig. 9:**
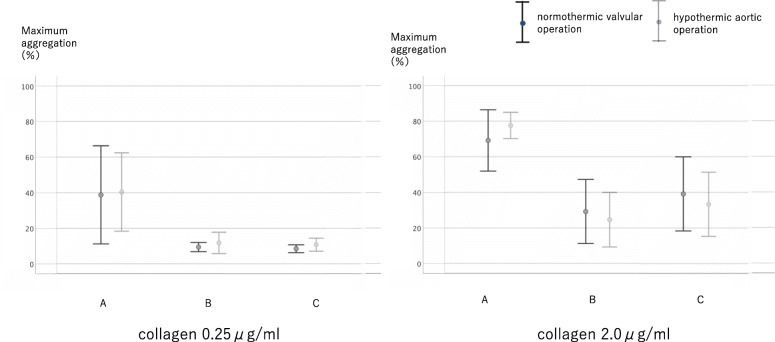
Change of platelet aggregation ability using collagen in normothermic valvular operation and hypothermic aortic operation. No significant differences were observed between the groups in terms of platelet aggregation ability during surgery

When the normothermic valvular surgery and hypothermic aortic surgery groups were compared, no differences were observed with regard to the platelet count (Fig. 7), aggregation ability (Figs. 8, 9) during surgery including autologous PRP collection and autologous PRP return.

## Discussion

The number of platelets reportedly decreases by 30–50% during extracorporeal circulation [5, 6]. The suspected causes include dilution by CPB introduction, damage due to flow in a nonphysiological environment, and activation of platelet aggregation [7]. Autologous platelet collection is a method for preservation of platelets before mechanical damage during CPB, which is returned to the patient after the CPB has been discontinued; it can avoid consumption due to platelet damage and activation [3, 8]. However, this method is limited in its usefulness, because it requires approximately 90 min for collection, after induction of anesthesia and prior to introduction of heparin.

Platelet function is reportedly inhibited by various antiplatelet drugs; however, inhibition of platelet function during administration of heparin has not been shown to occur other than in cases of heparin-induced thrombocytopenia (HIT) [9], in which antibodies promote the production of antiplatelet factor 4 heparin complex antibody, thereby promoting platelet aggregation [10].

There has been no study regarding the effects of heparin administration before CPB on platelet aggregation. We previously reported that there was no difference in autologous platelet aggregation between those collected before and after heparin administration in an animal model. We concluded that platelet count and platelet function in PRP are not affected by heparin administration [4]. Based on this experimental result, we planned the present collection of autologous platelets from blood after administration of heparin at the early stage of CPB initiation. The present results indicate that it is clinically feasible to collect autologous platelets from CPB circuit. This is consistent with the observation study which demonstrated that plasma components, platelet count, and platelet aggregation can be restored when plasma and platelets are sequestered at the time of CPB and subsequently returned after the procedure [11]. This autologous PRP collection from CPB circuit seems to be feasible and safe. Platelet count and aggregation ability had improved after the return of autologous PRP in this study. However, platelet count and aggregation ability significantly decreased after CPB including autologous PRP collection. It was unclear whether significant decrease of platelet count and aggregation ability is due to CPB, autologous PRP collection, or both. Autologous PRP collection may have the risk of bleeding tendency after CPB because of significant decrease of platelet count and aggregation ability. Further examination is necessary to clarify this concern.

The number of patients that anti-platelet drugs are given has increased because less invasive endovascular interventions are developed, and will increase in future. We have excluded the patients with preoperative antiplatelet drug use to evaluate accurate platelet aggregation ability in this study. Platelet aggregation ability had recovered after a certain period of cessation time. We have reported that the platelet aggregation ability had already recovered at 5 days after clopidogrel cessation [12].

Therefore, we think that autologous PRP collection is also useful for the patient with antiplatelet drug use.

Recently, a meta-analysis concerning the autologous PRP have been reported which mention its usefulness in surgery with CPB [8].

The present study was limited in that the subjects were not compared with a control group. Moreover, there was no indicator other than coagulation ability to accurately determine the extent of platelet damage by cardiopulmonary bypass; other indices are influenced by various factors. We will evaluate the usefulness of the autologous PRP collection from CPB circuit by means of comparison between the cases with autologous PRP collection and without autologous PRP collection in future.

## Conclusions

It was possible to collect autologous platelets with effective agglutination ability from the cardiopulmonary bypass circuit. Both blood platelet count and aggregation ability were increased by returning autologous PRP to patients after the discontinuation of cardiopulmonary bypass. This approach may represent an effective countermeasure to the shortage of blood products that our society is likely to encounter in the near future.

## Data Availability

The datasets used and analyzed during the current study are available from the corresponding author on reasonable requests.

## Abbreviations

PRP: Platelet-rich plasma
CPB: Cardiopulmonary bypass
ADP: Adenosine Diphosphate
HIT: Heparin-induced thrombocytopenia
